# Queer in STEM Organizations: Workplace Disadvantages for LGBT Employees in STEM Related Federal Agencies

**DOI:** 10.3390/socsci6010012

**Published:** 2017-02-04

**Authors:** Erin A. Cech, Michelle V. Pham

**Affiliations:** 1 Department of Sociology, University of Michigan, 500 S State St, Ann Arbor, MI 48109, USA; 2 Population Studies Center, University of Michigan, 500 S State St, Ann Arbor, MI 48109, USA

**Keywords:** STEM, LGBT inequality, Federal Agencies

## Abstract

Lesbian, gay, bisexual, and transgender (LGBT) individuals in U.S. workplaces often face disadvantages in pay, promotion, and inclusion and emergent research suggests that these disadvantages may be particularly pernicious within science and engineering environments. However, no research has systematically examined whether LGBT employees indeed encounter disadvantages in science, technology, engineering and math (STEM) organizations. Using representative data of over 30,000 workers employed in six STEM-related federal agencies (the Department of Energy, the Environmental Protection Agency, the National Science Foundation, NASA, the Nuclear Regulatory Commission, and the Department of Transportation), over 1000 of whom identify as LGBT, we compare the workplace experiences of LGBT employees in STEM-related federal agencies with those of their non-LGBT colleagues. Across numerous measures along two separate dimensions of workplace experiences—perceived treatment as employees and work satisfaction—LGBT employees in STEM agencies report systematically more negative workplace experiences than their non-LGBT colleagues. Exploring how these disadvantages vary by agency, supervisory status, age cohort, and gender, we find that LGBT persons have more positive experiences in regulatory agencies but that supervisory status does not improve LGBT persons’ experiences, nor do the youngest LGBT employees fare better than their older LGBT colleagues. LGBT-identifying men and women report similar workplace disadvantages. We discuss the implications of these findings for STEM organizations and STEM inequality more broadly.

## 1. Introduction

For decades, scholars have documented interactional- and institutional-level processes that perpetuate disadvantages for women and racial/ethnic minorities in science, technology, engineering, and mathematics (STEM). Although these professional arenas are increasingly committed to equality and inclusion [1,2], women and racial/ethnic minorities continue to face marginalization and discrimination in K-12 and college STEM education [3–8] and in STEM workplaces [5,9,10].

Do lesbian, gay, bisexual, and transgender (LGBT) persons experience similar forms of disadvantage within STEM-related environments? Gender and sexuality scholars have argued that hostility toward non-heterosexual identities and non-binary gender expressions often accompanies social contexts dominated by hegemonically masculine gender performances [11–13]. Such biases are rooted in beliefs about *gender* roles; they are reactions to what are presumed to be “normal” or “natural” expressions of gender identity and proper relationships between men and women [12–15]. Emergent research suggests that anti-LGBT bias may be particularly strident in STEM-related environments compared to other professional setting (e.g., [16–18]). As a set of professional arenas that are culturally dominated by hegemonically masculine-typed behavioral norms and interactional styles and that devalue femininity [7,10,19–21], STEM environments likely harbor *heterosexism*, bias and discrimination against LGBT persons that includes marginalization, harassment, and the denial of resources, and *heteronormativity*, a cultural schema that promotes an essentialized male/female sex binary and designates heterosexuality as the only normal sexuality [22,23].

Despite early exploration, researchers have yet to systematically determine whether LGBT workers encounter widespread disadvantages in their day-to-day experiences in STEM-related workplaces. The goal of this paper is to do just that. We use unique, representative survey data to compare the workplace experiences of LGBT employees to their non-LGBT colleagues and to explore whether these inequalities vary by agency, supervisory status, age, and gender. Drawing on ten distinct measures along two different dimensions of workplace experiences—treatment as employees and job satisfaction—this research offers a significant advancement in scholarly understanding of the contours of LGBT inequality in STEM environments.

We study what are, in many ways, “best-case scenario” organizations for LGBT equality and inclusion in science and engineering: STEM-related federal agencies. Unlike the private sector, which is at the whim of organizational, local, and/or state-level anti-discrimination policies, federal agencies have included sexual minorities in their non-discrimination policies since 1998 and transgender persons since 2012. Although heavily hierarchical and bureaucratized accountability structures are not always beneficial for the career advancement of under-represented groups [24], federal agencies are generally recognized as employing organizations with better average diversity outcomes [25–27] and greater equality in leadership [28] and remuneration [29] than organizations in the non-academic private sector.^1^ As such, patterns of inequality that we identify within these STEM-related federal agencies are likely present—if not amplified—in STEM organizations in the private sector. Additionally, these are powerful and important organizations for the safety and vitality of the U.S.; thus, it is especially important that all workers in these federal agencies are able to engage and contribute at work to their fullest capacity, regardless of their sexual identity and gender expression.

Six federal agencies are included in our sample: the National Aeronautics and Space Administration (NASA), the National Science Foundation (NSF), the Environmental Protection Agency (EPA), the Department of Energy (DOE), the Nuclear Regulatory Commission (NRC), and the Department of Transportation (DOT). These agencies are described in more detail below. A representative sample of employees from these agencies was included in the U.S. Office of Personnel Management’s 2013 Federal Employee Viewpoint Survey (FEVS). FEVS is, to our knowledge, the only large representative workplace survey that includes an LGBT self-identification measure and a range of workplace experience questions. Using these data, we are uniquely positioned to examine workplace experience inequalities by LGBT status in STEM-related agencies.^2^

Unlike much past research on inequality in STEM, ours is an examination of all employees in STEM-related organizations, not an analysis of the experiences of STEM professionals specifically. Workers with STEM training are employed in a great variety of organizations across the labor force—some of which are centrally focused on science and engineering tasks and others (e.g., healthcare or entertainment-related organizations) that are not centrally STEM-focused. The overall culture of an organization can be powerfully shaped by the cultural norms and values of the professional occupations which serve as the raison d’être of that organization [10]. NASA centrally involves aerospace science and engineering; EPA involves environmental and biological sciences; NSF evaluates and funds basic science, engineering, and math research; NRC is heavily involved with nuclear science and engineering; the DOT is centrally tasked with civil, transportation, and logistics engineering and technology; and the DOE promotes energy sciences. As such, the experiences of employees in these agencies is fundamentally shaped by the cultural norms and practices of STEM in ways that impact their day-to-day workplace experiences, even if employees themselves are not scientists, engineers, mathematicians, or technologists.

As a result of the systemic heterosexism and heteronormativity suggested by previous research on STEM environments [19,31], we expect that LGBT employees in these STEM-related federal agencies will report more negative treatment as employees and be less satisfied with their jobs than their non-LGBT colleagues. We expect that these patterns of inequality may vary depending on the context of each agency. In particular, agencies that principally serve a regulatory function may attract a slightly more politically liberal set of employees who may also be more supportive and inclusive of LGBT colleagues. We also explore whether these inequalities are mitigated for LGBT employees who have advanced in the hierarchy of their organization (i.e., are supervisors), whether recent cultural shifts toward greater rights and inclusivity for LGBT persons manifests as cohort differences in reported workplace experiences, and whether LGBT disadvantages play out differently by gender.

We find that LGBT employees report significantly more negative workplace experiences in these agencies than their non-LGBT colleagues across a number of workplace experience measures. These inequalities appear to be slightly less pronounced in the two regulatory agencies—the EPA and the NRC—relative to the other STEM-related agencies. Contrary to expectations, however, we do not find that inequalities are mitigated for LGBT employees who hold a supervisory role in their agency, nor for the youngest workers, suggesting that these workplace experience disadvantages may neither “get better” with career advancement nor disappear as younger generations of LGBT persons enter the workforce. Finally, we find that both men and women who identify as LGBT face similar workplace experience disadvantages in these agencies.

Examining LGBT inequality as a workplace dynamic is an important approach given the nature of LGBT status biases. Unlike other status characteristics such as gender or race/ethnicity, LGBT status is not reliably visible; LGBT status biases may operate within workplaces even when workers do not know or are not frequently reminded of the LGBT status of their co-workers [32,33]. As such, it is especially useful to study LGBT inequality by examining its entrenchment within STEM-related organizations.

## 2. Background

Recent research has identified a variety of ways that heterosexism and heteronormativity operate within the U.S. workforce. More than half of U.S. states lack employment discrimination legislation that includes LGBT status and several states are actively seeking to walk back such laws [34]. Within employing organizations, LGBT persons face formal and informal discriminatory policies such as health benefits that exclude transgender persons and same-gender domestic partners [22,35,36]. Informally, LGBT employees frequently encounter wage inequalities, social isolation from colleagues, workplace experience disadvantages, and pressures to downplay or cover their LGBT status (Author cite, [15,37,38]).

Emergent scholarship suggests that these LGBT inequalities are similar, if not more exaggerated, in STEM organizations. Exploratory qualitative work on LGBT employees in STEM organizations has found that they often are isolated from colleagues and feel they work harder than their non-LGBT colleagues to convince others of their competence as STEM professionals [16,39]. Studies of academic settings have found that LGBT students and faculty in STEM are more likely than LGBT persons in other disciplines to report discomfort with the campus climate and fear harassment and physical violence on campus [18,22,40,41].

Despite this early research, we know very little about the experiences of LGBT-identifying workers in science and engineering environments. This paper seeks to fill this void by comparing LGBT and non-LGBT colleagues in the same STEM-related organizations across an array of workplace experiences. As we discuss in the conclusion, the results presented here are likely applicable to STEM organizations across the labor force—even organizations that, like the federal agencies we study, have non-discrimination policies that include sexual identity and gender expression.

### 2.1. STEM-Related Federal Agencies

Six federal agencies are included in our study. To provide context for our analysis, we briefly describe the origins and goals of each agency.

#### Environmental Protection Agency (EPA):

Sparked by the physical and political fallout of the Santa Barbara oil spill in 1969, president Nixon formed the Environmental Protection Agency (EPA) in 1970 [42,43]. The EPA is a regulatory agency whose mission it is to protect human health and the environment and to “ensure compliance with environmental laws passed by Congress, state legislatures and tribal governments” [44]. The EPA works to make the United States air, water, and land cleaner and safer through policies such as the Clean Water and Clean Air Acts [45].

#### Nuclear Regulatory Commission (NRC):

In the wake of the first atom bomb detonation in 1945 and the Cold War arms race that ensued, the federal government saw a need to regulate nuclear materials and find civilian uses for nuclear energy. The Atomic Energy Commission (AEC) was created in 1946 to regulate and promote nuclear power. However, the AEC depended on the nuclear industry to produce data for them and to regulate themselves [46,47]. To address the inherent conflict of both promoting and regulating the nuclear industry, the 1974 Energy Reorganization Act split the AEC into the Nuclear Regulatory Commission (NRC) and the Energy Research and Development Agency (ERDA) [48].^3^ The NRC now oversees the nuclear industry by regulating nuclear materials and creating and enforcing nuclear safety requirements [47].

#### Department of Energy (DOE):

The energy shortage of the 1970s demonstrated the need for federal policy regarding energy creation and transmission, which had largely been left to the private sector [50,51]. President Carter signed the Department of Energy into action in 1977 [52]. The new agency’s responsibility was to “[advance] the national, economic, and energy security of the United States; [promote] scientific and technological innovation in support of that mission; and [ensure] the environmental cleanup of the national nuclear weapons complex” [50]. The Department of Energy is responsible for research and development of energy technologies, energy conservation and regulation planning, and energy data collection and analysis [48]. Currently, clean energy research and development stands as the DOE’s highest priority [53].

#### National Aeronautics and Space Administration (NASA):

Russia’s launch of the satellite Sputnik sparked a fervor in the U.S. government for aerospace research [54–56]. President Eisenhower signed the National Aeronautics and Space Act on 29 July 1958 to establish an agency that would “pioneer the future in space exploration, scientific discovery and aeronautics research” [56,57]. Building on the early accomplishments of its Apollo program, NASA is currently responsible for continued space and exploration research and works alongside the space programs of other nations and the nascent for-profit space exploration industry [57].

#### National Science Foundation (NSF):

Congress signed a bill in 1950 to establish the National Science Foundation, an agency intended to facilitate the perpetuation of the wartime pace of scientific and engineering advancements during times of peace [58]. The NSF’s central goals are to support research and education in science and engineering [59]. From its inception, the mission of NSF has dictated that scientists and engineers be in charge of the agency and that patenting guidelines are developed and managed by the foundation itself [60,61].

#### Department of Transportation (DOT):

The transportation infrastructure played an important role in the post-World War II economic boom [62]. By the 1960s, billions of dollars were being spent across eleven transportation-related agencies that dealt with differing facets of transportation management [62]. Congress adopted the Department of Transportation Act in 1966 to consolidate these efforts into one agency [63]. The new agency was intended to “serve the United States by ensuring a fast, safe, efficient, accessible and convenient transportation system that meets our vital national interests and enhances the quality of life of the American people, today and into the future” [64]. DOT is currently responsible for creating and coordinating wide-reaching transportation policies and programs across the U.S. [64].

Although the climate for LGBT-identifying employees may vary across these agencies, they—like all federal employees—are protected by anti-discrimination policies that are inclusive of sexual minority and gender expression. Furthermore, each of these agencies has an LGBT-specific Employee Resource Group (ERG) that provides networking, advocacy, and social support for LGBT employees and allies.

### 2.2. Hypotheses

Based on research discussed above on the experiences of LGBT persons in the workforce generally and in STEM specifically, we expect that LGBT-identifying employees in STEM-related federal agencies will report significantly less positive workplace experiences than their non-LGBT colleagues. We focus on two specific dimensions of workplace experiences, shown to be important in previous research on LGBT workplace inequality [32,65–67]: respondents’ perceived treatment as employees (e.g., whether they feel like their work is respected and that they are supported by their co-workers) and their work satisfaction (e.g., whether they are personally satisfied with their job and the extent to which they feel empowered at work). Due to processes of heterosexism and heteronormativity discussed above, we expect that LGBT persons will report significantly less positive treatment as employees, and be less satisfied with their work compared to their non-LGBT colleagues.

**Hypothesis 1:** *LGBT-identifying employees will report worse workplace experiences than their non-LGBT colleagues, net of agency, gender, racial/ethnic minority status, age cohort, tenure, and supervisory status*.

Support for LGBT equality and inclusion varies drastically across the two dominant political parties in the U.S. As entities that work in and around partisan politics, we might expect that some of this partisanship might play out within the context of the federal agencies themselves. However, as federal agencies, the six organizations that we study are bipartisan by definition and decree. The top-level leadership of several of them is purposefully comprised of an equal balance of democratic and republican appointed executives.

Although none of them are “conservative” or “liberal” agencies with corresponding views on LGBT equality, we suspect that the average workplace experiences for LGBT employees may vary depending the politicization of the core work of the agency. Specifically, there may be differences between the experiences of LGBT employees who work in agencies that principally serve a *regulatory* function (i.e., EPA and NRC), versus those that work in agencies that serve a number of other functions. Government regulation *itself* is politicized: conservative political leaders and legislators have called for broad-scale deregulation, and conservative voters tend to be deeply unsupportive of expanding government regulation [68,69].^4^ As such, we expect that the regulatory agencies in our sample may attract a slightly more politically and socially liberal set of employees than the other agencies. These more liberal pro-regulation employees may thus be more supportive of LGBT rights and inclusion [71,72] than the average employees in other federal agencies.

**Hypothesis 2:** *LGBT employees who work in regulatory agencies (i.e., EPA and NRC) will report more positive workplace experiences than LGBT employees who work in other STEM-related agencies, net of age cohort, supervisory status, gender and racial/ethnic minority status*.

Previous research on LGBT persons in the workforce has suggested that those who occupy leadership positions in their organizations have more social power and are more able to be open about their LGBT status than those who are lower in the organizational hierarchy and thus more vulnerable [65,66].^5^ As such, we expect that LGBT persons who are supervisors may be comparatively insulated from the effects of LGBT status biases and report significantly more positive workplace experiences compared to LGBT persons who do not hold a supervisory role in their organization:

**Hypothesis 3:** *LGBT employees who are supervisors report significantly more positive workplace experiences than their LGBT colleagues who are not supervisors, net of agency, age cohort, tenure, gender and racial/ethnic minority status*.

Over the last several decades, public views on sexual minorities and transgender individuals have changed dramatically [73]. Although over half of Americans express some level of disapproval toward sexual minorities [74], younger cohorts have entered the workforce during a time when blatant heterosexism is on the decline and state and local legislation has become more equitable overall for LGBT-identifying persons. Older LGBT-identifying federal employees experienced a different work environment in past decades. Up until the 1980s, LGBT persons were regularly denied the security clearances so often required of work in science and engineering agencies and were subject to invasive questioning in security clearance applications through the 1990s [75]. Given these political and social changes, we might expect that the youngest cohorts of LGBT-identifying workers may report systematically more positive work experiences than their older colleagues. Even if older LGBT colleagues face a qualitatively better work environment now than in the past, their views on their current workplace experiences may be colored by past experiences of prejudice. As such, we expect there to be a significant and negative interaction effect between age cohort and LGBT status, meaning that younger LGBT persons have more positive workplace experiences than older LGBT colleagues.

**Hypothesis 4:** *Older LGBT employees will report significantly more negative workplace experiences than younger LGBT employees, net of agency, gender, racial/ethnic minority status, tenure, and supervisory status*.

If, however, the LGBT * age cohort interaction terms are insignificant, this would indicate that younger cohorts of LGBT persons are not generally better off than their older LGBT colleagues.

Finally, we examine whether these LGBT inequalities vary by gender. Existing scholarship does not suggest an obvious set of relationships. For men, LGBT status likely undermines workplace experiences, both because of negative status biases toward LGBT persons in general and the cultural association of gay, bisexual, and transgender men with femininity [22] that is devalued in STEM environments [19]. The pattern among women is less clear. On the one hand, lesbian, bisexual, and transgender women are culturally associated with masculinity, which may mean that they are taken more seriously in STEM-related environments and have better work experiences [19]. On the other hand, LGBT-identifying women’s divergence from normative or “natural” gender roles may mean that they encounter more negative treatment and have less work satisfaction than other women in their agencies. We investigate whether there are gendered patterns in these LGBT inequalities by comparing LGBT and non-LGBT men and women separately. The results of this analysis shed light on gender processes in STEM environments more generally by helping to disentangle whether gender biases are principally an issue of the devaluation of *femininity* (which would reflect LGBT biases for men but no effect—or possibly a benefit—of LGBT status for women) or broader processes related to the norms and expectations of the gender structure more broadly.

## 3. Methods

We use data from the 2013 Federal Employee Viewpoint Survey (FEVS) for these analyses. FEVS is a representative survey of employees in federal agencies in the U.S. conducted by the Office of Personnel Management (OPM). In 2012, OPM added a question about LGBT status to the FEVS. Although FEVS has limitations in that it is cross-sectional and does not (for reasons of confidentiality) include details on respondents’ occupation or specific positions of employment, it is the only available dataset that offers the ability to assess the workplace experiences of LGBT persons in STEM-related organizations using representative data.

The full 2013 FEVS sample contains 376,577 employees.^6^ For this analysis, we use the 37,219 respondents who are employed in the six STEM-related agencies. We use multiple imputation (20 multiply-imputed datasets) to handle missing data on all measures except LGBT status^7^ and all models are weighted with the OPM-provided proportional weight “postwt”.

### 3.1. Variable Operationalization

Table 1 provides information on specific question wording and scale construction. LGBT status, the focal independent variable, was created by OPM out of a question that asked respondents: “Do you consider yourself to be one of the following (mark all that apply):” “Heterosexual or Straight”, “Bisexual”, “Gay or Lesbian”, “Transgender”, and “Prefer not to say”. Respondents who selected bisexual, gay or lesbian, or transgender were coded as LGBT. Respondents who marked “prefer not to say” (12% of the sample) were excluded from the analysis.

We examine two dimensions of workplace experiences: treatment as employee measures and work satisfaction measures (also see [32]. The range of measures we include are important: some of them (e.g., job satisfaction, respected by supervisor) are less dependent on one’s particular occupation or hierarchical position than others (e.g., adequate resources). This range also allows us to understand whether patterns of inequality seem to coalesce around only one type of workplace experience inequality or extend across a wider array of issues.

The individual measures, and the variables that were used to make up the scales, are detailed in Table 1. In the original FEVS instrument, respondents were asked their level of agreement with each statement on a 1–5 scale (1 = “strongly disagree”, 2 = “disagree”, 3 = “neither agree nor disagree”, 4 = “agree”, and 5 = “strongly agree”). Questions related to work satisfaction were asked with a parallel 1–5 scale ranging from “very dissatisfied” to “very satisfied”. In order to help protect confidentiality, OPM recoded the 1–5 response values on each question into a 1–3 positive/negative response range, where 3 = positive (agree or strongly agree; satisfied or very satisfied), 2 = neutral (neither agree nor disagree; neither satisfied nor unsatisfied), and 1 = negative (strongly disagree or disagree; very dissatisfied or dissatisfied). The index measures below were divided by the number of measures in the index in order to retain a 1–3 value range.

### 3.2. Controls

Our models control for as wide a range of demographic and employment variables as is available in the data. Specifically, we control for gender (1 = woman, 0 = man), racial/ethnic minority (REM) status (1 = identify as African-American, Asian, Hispanic or Latino, Native American and/or other nonwhite identity; 0 = identifies as white, non-Hispanic), tenure in one’s agency (1 = 5 or fewer years, 2 = 6–14 years, 3 = 15 or more years), supervisory status (1 = supervisor, manager, or executive; 0 = non-supervisor or team leader), and age cohort (1 = under 40 years; 2 = 40–49 years; 3 = 50–59 years; 4 = 60 years or above). Due to concerns about possible identifiability and loss of confidentiality, the Office of Personnel Management does not provide educational background level or occupational category in FEVS, nor does it grant restricted-use access to such data. As such, we are not able to determine whether individual respondents in these agencies are employed in STEM occupations or have STEM degrees.

### 3.3. Analytic Strategy

Means and standard errors for all respondents and for LGBT and non-LGBT persons separately are included in Table 2. To test our hypotheses, we use OLS regressions for all but one of our dependent measures. Because overall job satisfaction is a single-question measure with a 1–3 value range, we use an ordered logistic regression model for that measure. We test the first hypothesis with models that include the LGBT identity measure, alongside controls for gender, REM status, supervisor status, employment tenure, age cohort, and agency. For the second hypothesis, we re-run this set of models with interaction terms between LGBT status and each of the agencies, including these interaction terms in the models one at a time. Hypotheses 3 and 4 are tested by adding to the previous models interaction terms for LGBT status X supervisor status and LGBT status X age cohort, respectively. Finally, to investigate possible differences in the effect of LGBT status by gender, we present the LGBT coefficients for each outcome measure in models ran separately for men and women.

## 4. Results

Table 2 provides means and standard errors for the demographic and workplace experience measures for all respondents and separately by LGBT status. Just under three percent (2.8%) of our sample identifies as LGBT. This is noticeably lower than national estimates that 3.4% of the U.S. population identifies as LGBT [65], suggesting that LGBT persons are under-represented in these STEM-related agencies relative to their representation in the U.S. population in general. Compared to non-LGBT respondents, there is a significantly lower proportion of REMs among the LGBT sample and the LGBT sample is typically younger and has a shorter tenure. In these bivariate statistics, LGBT persons have significantly more negative workplace experiences across all of the measures included here. The multivariate analyses below will test whether these differences remain net of variation in age, tenure, supervisory status, gender and race/ethnicity. Table 2 also provides the proportion of the total sample are employed in the six different agencies. Appendix A Table A1 provides the representation of LGBT persons in each agency, ranging from a high of 5.0% at the EPA and a low of 2.2% at NASA.

We hypothesized above that LGBT-identifying employees would report worse workplace experiences than their LGBT colleagues in the form of more negative treatment and worse job satisfaction. Table 3 presents the results of the OLS and ordered logit models predicting the workplace experience measures with LGBT status and controls. As expected, LGBT-identifying employees report more negative workplace experiences along a variety of different measures: they are less likely than their non-LGBT colleagues to report that their success is fostered, that they have adequate resources, that their organization supports diverse workers, and (marginally) that they have transparent evaluations in their workplace. We also find substantial differences in job satisfaction: LGBT employees report significantly lower satisfaction with employee empowerment and organizational procedures in their agency, and lower overall job satisfaction than their non-LGBT colleagues. They are also marginally significantly less likely to report personal satisfaction with their work and satisfaction with the working conditions in their organization. On average, these effect sizes are about a tenth of a point on a three-point scale (1 = negative to 3 = positive).

**Figure 1.**
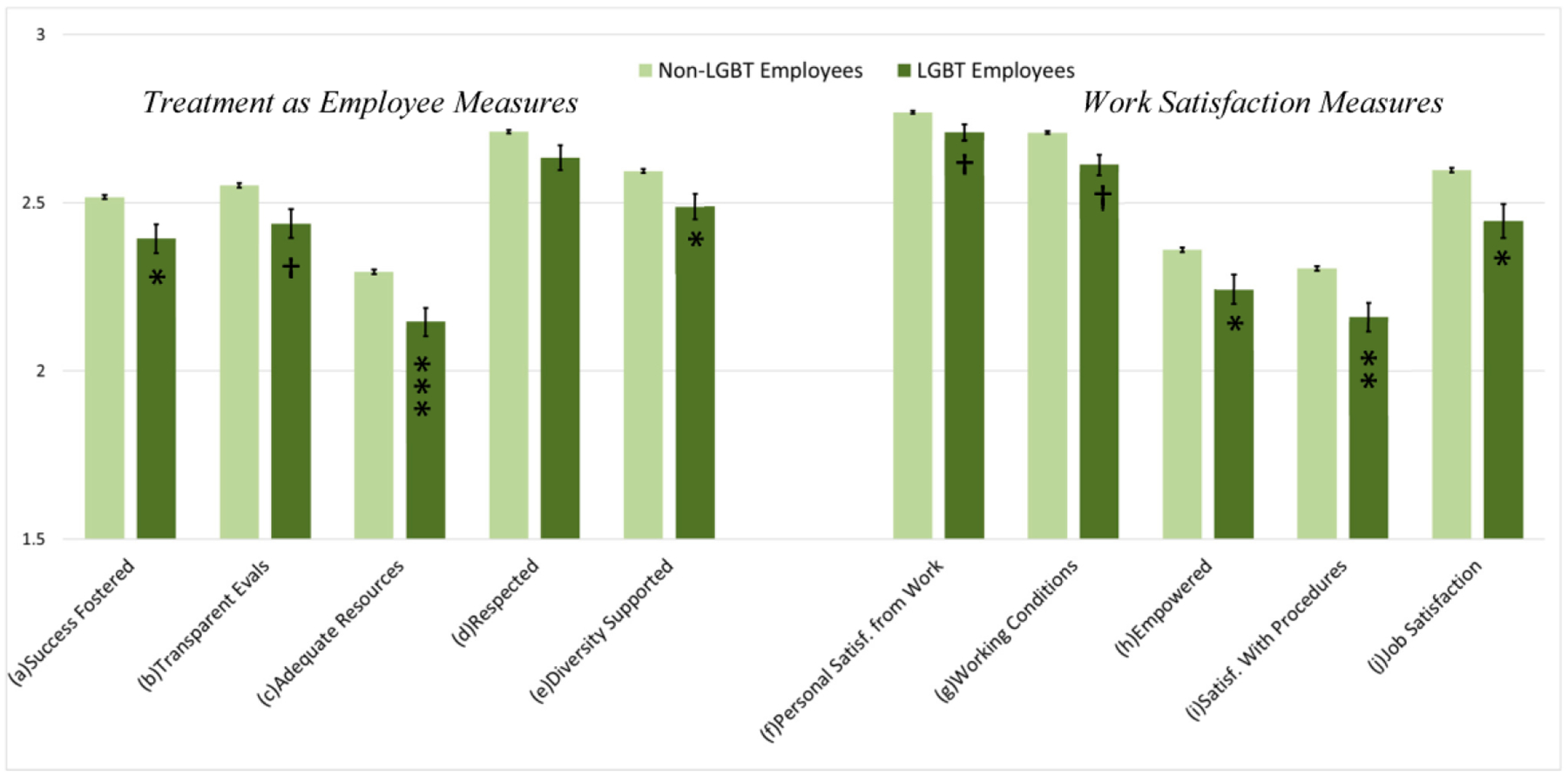
Workplace Experiences among Non-LGBT and LGBT Employees. *Height* of the columns indicates the means for LGBT and non-LGBT employees, respectively (error bars = 95% C.I.s). *Asterisks* indicate significance of LGBT status net of variation by gender, REM status, tenure, age category, and agency (* *p* < 0.05, ** *p* < 0.010, *** *p* < 0.001, based on two-tailed tests; 1 = negative, 2 = neutral, 3 = positive). See Table 3 for significance levels of the direct comparison of LGBT and non-LGBT workers.

Overall, these results indicate workplace experience inequalities for LGBT-identifying employees across a wide range of treatment and satisfaction measures (supporting H1). Figure 1 presents the mean values on each workplace experience measure for LGBT and non-LGBT workers, respectively. Error bars represent 95% confidence intervals (±1.96×SE). The asterisks indicate the significance of the difference between LGBT and non-LGBT coworkers on each measure, net of controls (significance values taken from Table 3).

Although the STEM-related federal agencies in our sample share anti-discrimination regulations and procedures, we expect there to be variation in the experiences for LGBT persons across these agencies. In particular, we expect that LGBT employees will do slightly better in agencies that served a primarily regulatory purpose, as workers with conservative anti-regulation political views (which are correlated with less positive views of LGBT equality) may be likely to self-select out of those agencies.

**Figure 2.**
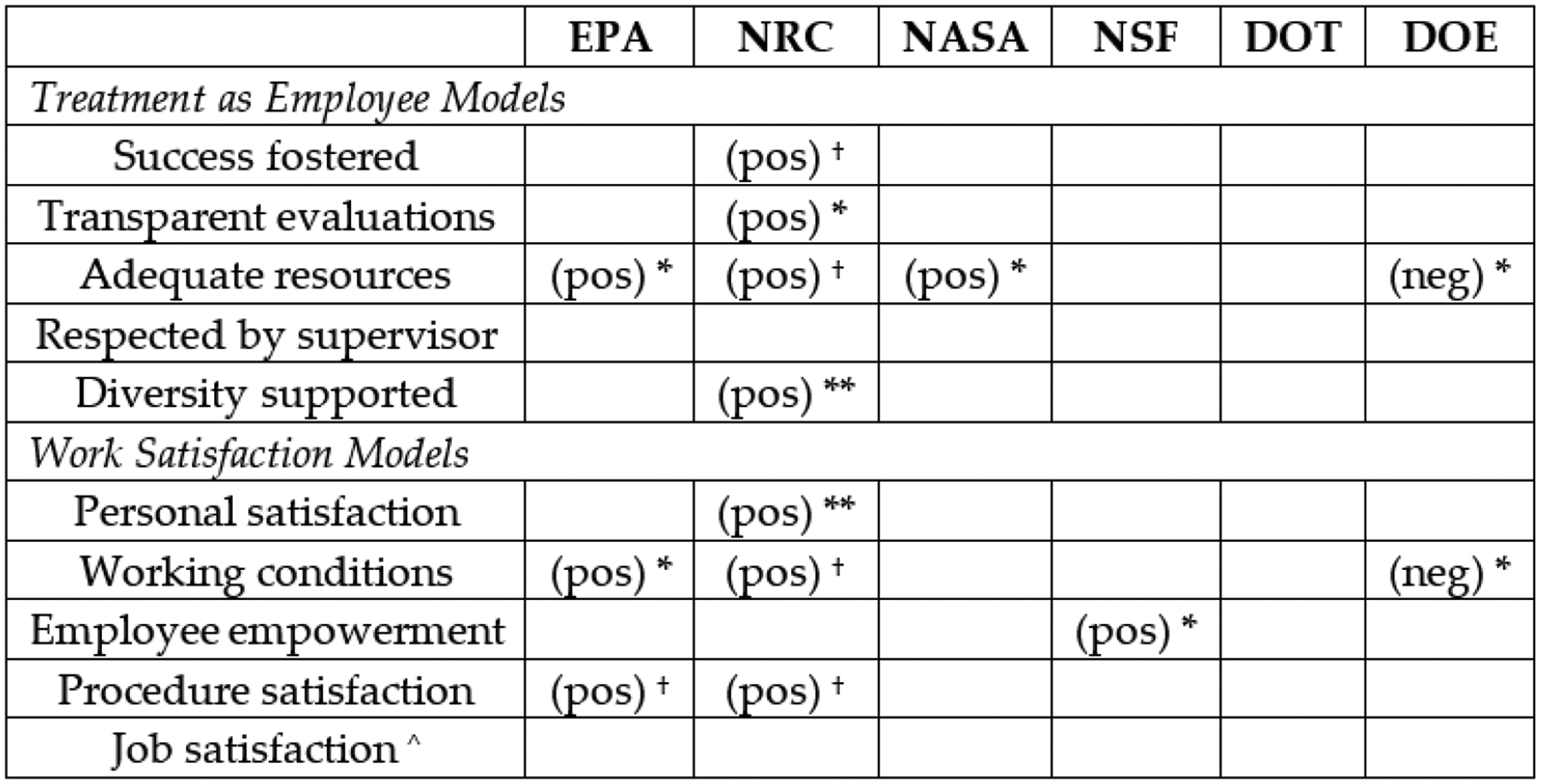
Significance Level and Direction of LGBT*Agency Interaction Terms, for Regulatory (EPA, NRC) and Non-Regulatory (NASA, NSF, DOT, DOE) Agencies. Note: ^†^ *p* < 0.10, * *p* < 0.05, ** *p* < 0.01, *** *p* < 0.001 (two-tailed test). Only significant interaction terms are presented; all other interactions terms have *p* > 0.10. ^ indicates ordered logit model; all other models are ordinary least squared (OLS) regressions. Coefficients, standard errors and p-values for each coefficient are presented in Appendix Table A1.

To test Hypothesis 2, we predicted the workplace experience measures with an interaction term between LGBT status and each agency indicator. The LGBT*agency measures were included in the models one at a time. Figure 2 summarizes the patterns of significance for these interaction terms. Specifically, the figure presents the direction and level of significance of the interaction term between that agency and LGBT status that reach at least marginal statistical significance (*p* < 0.10). A significant and positive interaction term would indicate that LGBT respondents at that agency have significantly more positive experience on that measure compared to LGBT respondents in other agencies. Appendix A Table A2 presents the coefficients, standard errors, and *p*-values for each of these interaction terms.

The results are in the expected direction: LGBT respondents employed in the EPA are significantly more likely to report that they have adequate resources and to be satisfied with their working conditions, and marginally more likely to report satisfaction with organizational procedures, compared to LGBT persons in the other STEM-related agencies. The Nuclear Regulatory Commission also promotes more positive workplace experiences for LGBT persons compared to other agencies along several dimensions: LGBT persons in the NRC report more transparent evaluations, more support for diversity, and, marginally, more adequate resources and more satisfaction with working conditions and procedures, compared to LGBT employees in other agencies. NSF LGBT employees reported significantly higher levels of employee empowerment and NASA LGBT employees reported greater resources than LGBT employees at other agencies. On the flip side, LGBT employees in the DOE are significantly less likely to report adequate resources and more negative working conditions than LGBT employees at other agencies.^8^

An alternative explanation for the generally more positive patterns at the NRC and the EPA might be that they are demographically different than the other agencies—that they have greater representation of LGBT employees, greater gender and race diversity, or their workforce is younger and thus potentially more accepting of LGBT persons than the workforce at NASA, NSF, DOE and DOT. Appendix A Table A1 provides employee demographics broken down by agency. The NRC is not an outlier in its demographic diversity nor the average age of its employees. The EPA has the highest proportion of LGBT employees (5 percent), which may help improve the experiences of LGBT persons overall in that agency [32], but has comparatively low representations of women and people of color and employees of similar average age. As such, the demographic contours of NRC and EPA do not appear to be the driving factor in the patterns documented in Figure 2.

Beyond agency differences, we hypothesized that supervisory status may insulate LGBT persons from some of the disadvantages that those lower in the organizational hierarchy encounter (H3). Table 4 presents the coefficients, p-values, and significance of the interaction term between LGBT*supervisory status. Contrary to our hypothesis, we find that none of the interaction terms are significant and all are substantively small. This suggests that the workplace experience inequalities that LGBT employees face in these agencies are not lessened among those who have obtained supervisory status. In contrast, the main effect for the supervisor indicator, which now represents results from non-LGBT supervisors, is strong and positive for most workplace experience measures, suggesting that non-LGBT persons have generally more positive workplace experiences when they are supervisors. The same does not hold for LGBT employees.

Next, we hypothesized that, consistent with substantial cultural and legislative shifts toward LGBT equality and inclusion, younger LGBT employees would report more positive workplace experiences than their older LGBT colleagues (H4). However, there is little indication of age cohort effects: all but one of the interaction terms between LGBT status and age cohort are non-significant (Table 5).^9^ We discuss the implications of these results in the next section.

Finally, we tested whether there are gender differences in the manifestation of LGBT inequality. Table 6 presents LGBT coefficients for models ran separately for men and women. We find similar patterns across both sets of models. This is confirmed by supplemental analyses (not shown) replicating the models in Table 3 with LGBT*woman interaction terms; none of the interaction terms were significant. This runs counter to possible expectations that LGBT-identifying women may experience less workplace experience disadvantages than non-LGBT women because of the cultural assumptions that non-heterosexual and transgender women are more masculine than heterosexual women. It indicates that both men and women who identify as LGBT face similar workplace experience disadvantages.^10^

## 5. Discussion

The goal of this paper is to examine whether there are significant workplace experience inequalities for LGBT-identifying employees within STEM-related agencies and whether those inequalities vary by agency, worker supervisory status, worker age, and gender. As hypothesized, we found evidence of widespread workplace experience inequalities for LGBT employees compared to their non-LGBT colleagues. We found these inequalities to be somewhat lessened—but not completely mitigated—in agencies with primarily regulatory missions and goals. Although we cannot say for certain that the regulatory focus of these agencies produces a selection effect that deters politically conservative employees, the results for the EPA and NRC are in line with these expectations. Supplemental analyses also helped rule out demographic diversity and average age and tenure as possible explanations.

These first two sets of results have important implications. First, LGBT workplace experience inequalities appear to be quite widespread within STEM-related agencies. We find significant differences by LGBT status on a variety of workplace experience inequalities ranging from the lower likelihood of reporting that their success is fostered and they have adequate resources, to their perception of a lack of support for diversity, to lower job satisfaction.

Additionally, our results suggest that the particular socio-political context of the organization may have consequences for the experiences of LGBT persons, even if the point of politicization (in this case, regulatory functions) is not directly connected to LGBT equality issues. As noted in the introduction, antidiscrimination policies and employee benefits are held constant across these six federal agencies. The politicization of the work of other non-governmental STEM-related organizations (e.g., defense contractors or companies that use stem cells for biomedical research) may, by the nature of their central work tasks, attract employees that tend to be more or less supportive of LGBT equality and inclusion. More research is needed to understand how particular organizational focus and goals can promote more positive or negative workplace experiences for LGBT workers.

Our analysis also produced insightful null findings. First, counter to our expectations, supervisory status does not appear to insulate LGBT employees from negative workplace experiences. LGBT supervisors do not fare better than non-supervisors on any of the workplace experience measures. While supervisory status does provide workers with more authority and power within an organization, it does not appear to protect them from colleagues’ bias.

Second, we found that the youngest LGBT workers in these agencies do not have systematically more positive workplace experiences than their older LGBT colleagues. This is a striking finding, as employees in these agencies who were required to gain security clearance three decades ago would have had to remain closeted in order to keep that clearance [77]. Of course, the responses of older LGBT employees may simply reflect their more positive current workplace experiences compared to more egregious heterosexism and heteronormativity in the past, but the memories of past negative workplace experiences might color their view of their current workplace experiences, compared to younger workers who have enjoyed LGBT-inclusive non-discrimination policies and a more tolerant cultural landscape for the entirety of their (short) careers. This suggests that the resolution of these LGBT inequalities is not simply a matter of waiting for this to “get better” as younger generations of LGBT persons enter the workforce their older colleagues retire.

Third, we find that there is little difference in the reported experiences of LGBT-identifying women and men. One possible alternative explanation for the results of LGBT status in the full sample is that it is not LGBT status per se that is devalued, but *femininity* within the context of a culturally masculine organizations. In this perspective, the LGBT effects would be primarily driven by the devaluation of perceived femininity among LGBT-identifying men in STEM environments. However, our results indicate that LGBT-identifying women are similarly disadvantaged compared to non-LGBT women. This suggests that the LGBT results are rooted in status biases related to the normative expectations for “normal” or “natural” performances of gender rather than just the devaluation of femininity in a masculine space.

These analyses have several limitations worth noting. FEVS is a cross-sectional survey, so we are unable to follow workers over time as they encounter and react to their workplace experiences. Future work with longitudinal data would also be better able to discern how workers’ experiences change as they move into supervisory positions. Related, although we believe that the agency-specific effects are due at least in part to differences in politicized priorities that may lead to a selection of more politically liberal workers into regulatory agencies, we cannot rule out other possible explanations. Third, as the LGBT samples at some of the agencies were quite small (especially NSF and NRC), it is possible that there may be agency effects that were not picked up by the interaction terms.

Finally, the FEVS does not have data on respondents’ occupation; thus we cannot distinguish between workers who are engaged directly in STEM work and those who do other types of work (e.g., administrative or human resources) in these STEM-related agencies. It would have been particularly helpful to have occupation in order to control for possible patterns of occupational segregation in these results. Some of the workplace experience differences we see by LGBT status may be due in part to the under-representation of LGBT persons in occupations within the STEM-related organizations that have less power and prestige. This may explain part of the relationship between LGBT status and adequate resources and satisfaction with working conditions.^11^ For example, if LGBT employees are less likely to be in “line” positions working as STEM professionals in these STEM-related agencies, they may, by the nature of their jobs, have less adequate resources and less satisfaction with their working conditions on average than non-LGBT persons. However, most other measures (e.g., respected by supervisor, job satisfaction) are not so closely tied to occupation. To account for as much variation by job and occupation as possible, our models controlled for supervisory status and tenure.^12^ Despite these limitations, these data provide an unprecedented opportunity to examine the contours of LGBT inequality among a representative sample of workers in a number of STEM-related organizations. Because recent literature has begun to demonstrate that anti-LGBT bias can be particularly pernicious among STEM professionals, we expect that these results (which include STEM and non-STEM workers in STEM-related agencies) *underestimate* the workplace experience disadvantages LGBT-identifying STEM professionals encounter.

## 6. Conclusion

Overall, these results illustrate that LGBT workplace experience inequalities are pervasive within STEM-related agencies, extend across age cohorts and supervisory status, and exist for both LGBT-identifying women and men. This has several implications for STEM-related organization inside and outside the federal government. As we noted in the introduction, federal agencies have expansive non-discrimination policies and bureaucratized accountability structures that formally protect LGBT employees. Nevertheless, workplace experience inequalities for LGBT persons persist in these agencies. Although many high-profile STEM organizations in the private sector have promoted LGBT inclusion for decades, these protections are not industry-wide. As such, the inequalities we document here are likely present—if not exaggerated—in STEM-related organizations in the private sector. Additionally, previous research has illustrated that workplace satisfaction and more negative treatment can reduce worker engagement and productivity [78–80]. As such, the workplace experience inequalities documented here may actually serve to reduce the productivity and efficiency of these STEM organizations.

LGBT inequality is an informative axis of disadvantage to consider in STEM. Not only is LGBT status an important social category in its own right, but the results here suggest that consideration of LGBT status sheds light on gender inequality as well. Our results suggest that devaluation as a result of the norms of the gender structure—not just the devaluation of femininity—reproduces inequality in STEM environments. Further research is needed to discern how sexual identity and transgender status intersect with professional and organizational cultures in STEM and how these biases interface with gender biases documented in prior scholarship. Understanding how inequality is reproduced along a variety of demographic axes is the first step toward developing policies and practices that make STEM as inclusive as possible.

## Figures and Tables

**Table 1.** Operationalization of workplace experience dependent variables.

Perceived Treatment as Employees	
Work Success is Fostered	“I feel encouraged to come up with new and better ways of doing things”, “I am given a real opportunity to improve my skills in my organization”, “I have enough information to do my job well”, and “My talents are used well in the workplace”. (1 = [neg]ative to 3 = [pos]itive; α = 0.820)
Transparent Evaluations	“My performance appraisal is a fair reflection of my performance”, “My supervisor/team leader provides me with constructive suggestions to improve my job performance”, and “Discussions with my supervisor/team leader about my performance are worthwhile”, (1 = neg to 3 = pos; α = 0.816)
Adequate Resources	“I have sufficient resources to get my job done”, “My workload is reasonable”, and “My training needs are assessed”. (1 = neg to 3 = pos; α = 0.657)
Respected by Supervisor	“My supervisor/team leader listens to what I have to say”, “My supervisor/team leader treats me with respect”, and “My supervisor/team leader provides me with opportunities to demonstrate my leadership skills”. (1 = neg to 3 = pos; α = 0.852)
Diversity Supported	“My supervisor/team leader is committed to a workforce representative of all segments of society”, “Policies and programs promote diversity in the workplace”, “Prohibited Personnel Practices are not tolerated”, and “Managers/supervisors/team leaders work well with employees of different backgrounds”. (1 = neg to 3 = pos; α = 0.798)
Workplace Satisfaction	
Personal Satisfaction from Work	“I like the kind of work I do”, “My work gives me a feeling of personal accomplishment”, and “The work I do is important”. (1 = neg to 3 = pos; α = 0.723)
Satisfaction with Working Conditions	“Employees are protected from health and safety hazards on the job”, “Physical conditions allow employees to perform their jobs well”, “My organization has prepared employees for potential security threats”, and “I recommend my organization as a good place to work”. (1 = neg to 3 = pos; α = 0.659)
Employee Empowerment	“Creativity and innovation are rewarded”, “Employees have a feeling of personal empowerment with respect to work processes”, “Employees are recognized for providing high quality products and services”, and “Supervisors/team leaders in my work unit support employee development”. (1 = neg to 3 = pos; α = 0.844)
Satisfaction with Procedures	“How satisfied are you with the recognition you receive for doing a good job?” “How satisfied are you with your involvement in decisions that affect your work?” “How satisfied are you with your opportunity to get a better job in your organization?” and “How satisfied are you with the information you receive from management on what’s going on in your organization?” (1 = neg to 3 = pos; α = 0.835)
Overall Job Satisfaction	“Considering everything, how satisfied are you with your job?” (1 = neg to 3 = pos)

**Table 2.** Univariate and Bivariate Statistics for LGBT and non-LGBT Respondents.

	ALL		LGBT		Non-LGBT		*p*
	(N = 37,219)		(N = 1042)		(N = 36,177)		
	Mean	SE	Mean	SE	Mean	SE	
LGBT	0.028	0.001	n/a		n/a		
Female	0.369	0.003	0.379	0.016	0.369	0.003	
Racial/Ethnic Minority	0.279	0.002	0.251	0.014	0.279	0.002	*
Supervisor	0.184	0.002	0.183	0.012	0.184	0.002	
Age cohort	2.495	0.005	2.293	0.030	2.500	0.005	***
Tenure	2.318	0.004	2.219	0.025	2.321	0.004	***
*Treatment as Employee*							
Success fostered	2.513	0.003	2.394	0.021	2.517	0.003	***
Transparent evaluations	2.548	0.003	2.438	0.022	2.551	0.003	***
Adequate resources	2.291	0.003	2.146	0.021	2.295	0.003	***
Respected by supervisor	2.709	0.003	2.635	0.019	2.711	0.003	***
Diversity supported	2.592	0.003	2.489	0.019	2.594	0.003	***
*Work Satisfaction*							
Personal satisfaction	2.768	0.002	2.710	0.015	2.769	0.002	***
Working conditions	2.705	0.002	2.613	0.015	2.708	0.002	***
Employee empowerment	2.357	0.003	2.243	0.022	2.360	0.003	***
Procedure satisfaction	2.300	0.003	2.160	0.021	2.304	0.003	***
Job satisfaction	2.593	0.004	2.446	0.025	2.597	0.004	***
*Agencies*							
EPA	0.084	0.001	.152	0.011	0.082	0.001	***
NRC	0.053	0.001	0.053	0.007	0.053	0.001	
NSF	0.018	0.001	0.027	0.005	0.018	0.001	
NASA	0.218	0.002	0.168	0.012	0.220	0.002	
DOT	0.487	0.003	0.464	0.015	0.487	0.003	
DOE	0.140	0.002	0.136	0.011	0.140	0.002	

† *p* < 0.10,

* *p* < 0.05,

** *p* < 0.01,

*** *p* < 0.001 (two-tailed test comparing LGBT and non-LGBT respondents).

**Table 3.** OLS and Ordered Logistic Regression Models Predicting Workplace Experience Measures with LGBT Status and Controls (N = 37,219).

	Treatment as Employees Measures									
	Success Fostered		Transparent Evaluations		Adequate Resources		Respected by Supervisor		Diversity Supported	
	B	*S.E*.	B	*S.E*.	B	*S.E*.	B	*S.E*.	B	*S.E*.
LGBT	−0.115 *	0.046	−0.084^†^	0.043	−0.162 ***	0.046	−0.074	0.046	−0.147 *	0.066
Female	0.035 **	0.013	0.005	0.013	0.011	0.013	−0.031 *	0.014	−0.029 *	0.012
REM	0.008	0.015	−0.017	0.014	0.081 ***	0.015	−0.035 *	0.014	−0.134 ***	0.014
Supervisor	0.203 ***	0.014	0.130 ***	0.014	−0.048 **	0.016	0.153 ***	0.011	0.216 ***	0.011
Tenure	−0.060 ***	0.011	−0.075 ***	0.011	−0.066 ***	0.010	−0.041 ***	0.010	−0.079 ***	0.009
EPA	−0.018	0.017	0.067 ***	0.017	−0.173 ***	0.017	0.056 ***	0.015	0.058 ***	0.015
NSF	0.064 *	0.027	0.079 **	0.028	−0.088 **	0.029	0.029	0.026	0.037	0.025
NASA	0.267 ***	0.011	0.226 ***	0.012	0.177 ***	0.012	0.164 ***	0.010	0.246 ***	0.010
NRC	0.185 ***	0.015	0.149 ***	0.017	0.218 ***	0.017	0.111 ***	0.014	0.187 ***	0.014
DOT	0.004	0.015	0.071 ***	0.015	0.046 **	0.014	0.022	0.014	0.019	0.013
Constant	2.485 ***	0.027	2.577 ***	0.027	2.369 ***	0.025	2.750 ***	0.025	2.69 ***	0.022
	Workplace Satisfaction Measures									
	Personal Satisfaction		Satisfactory w/Working Conditions		Employee Empowerment		Procedures Satisfaction		Overall Job Satisfaction^^^	
	B	*S.E*.	B	*S.E*.	B	*S.E*.	B	*S.E*.	B	*S.E*.
LGBT	−0.049^†^	0.026	−0.080^†^	0.045	−0.115 *	0.046	−0.140 **	0.050	−0.379 *	0.153
Female	0.011	0.008	0.002	0.011	0.037 **	0.013	0.017	0.013	0.078^†^	0.044
REM	0.018 *	0.008	0.028 *	0.012	0.042 **	0.015	0.046 **	0.015	0.081^†^	0.048
Supervisor	0.098 ***	0.009	0.128 ***	0.011	0.274 ***	0.015	0.243 ***	0.016	0.505 ***	0.054
Tenure	−0.029 ***	0.006	−0.058 ***	0.008	−0.102 ***	0.011	−0.092 ***	0.010	−0.229 ***	0.035
EPA	−0.002	0.012	0.035 *	0.011	0.040 *	0.017	−0.037 *	0.016	−0.020	0.053
NSF	0.044 *	0.019	0.064 ***	0.017	0.057 *	0.028	0.003	0.027	0.105	0.093
NASA	0.096 ***	0.008	0.169 ***	0.008	0.386 ***	0.012	0.303 ***	0.011	0.695 ***	0.041
NRC	0.073 ***	0.012	0.152 ***	0.010	0.267 ***	0.017	0.233 ***	0.016	0.514 ***	0.061
DOT	0.059 ***	0.010	−0.014	0.011	0.009	0.015	0.030 *	0.015	0.279 ***	0.049
Constant	2.728 ***	0.015	2.686 ***	0.020	2.296 ***	0.027	2.280 ***	0.026	N/A	N/A

Notes: DOE is the comparator category for the agency. REM = Racial/ethnic minority status. Columns report unstandardized coefficients (and Std. Errors) from regression models.

† *p* < 0.10,

* *p* < 0.05,

** *p* < 0.01,

*** *p* < 0.001 (two-tailed test),

^ indicates ordered logit models; all other models are OLS regressions.

**Table 4.** OLS and Ordered Logistic Regression Models Predicting Workplace Experience Measures with LGBT X Supervisor Status Interaction Term (N = 37,219).

	Supervisor Coefficient	LGBT Coefficient	Supervisor * LGBT Coefficient	Supervisor * LGBT *p*-Value
*Treatment as Employee Models*				
Success fostered	0.203 ***	−0.115 *	0.004	0.957
Transparent evaluations	0.131 ***	−0.079	−0.041	0.572
Adequate resources	−0.049 ***	−0.169 **	0.046	0.574
Respected by supervisor	0.152 ***	−0.077	0.024	0.717
Diversity supported	0.215 ***	−0.151 *	0.026	0.768
*Work Satisfaction Models*				
Personal satisfaction	0.097 ***	−0.051^†^	0.020	0.670
Working conditions	0.128 ***	−0.079	−0.011	0.863
Employee empowerment	0.272 ***	−0.120 *	0.041	0.580
Procedure satisfaction	0.242 ***	−0.142 *	0.018	0.823
Job satisfaction^^^	0.506 ***	−0.376 *	−0.026	0.923

Note:

† *p* < 0.10,

* *p* < 0.05,

** *p* < 0.01,

*** *p* < 0.001 (two-tailed test).

^ indicates ordered logit model; all other models are OLS regressions.

**Table 5.** OLS and Ordered Logistic Regression Models Predicting Workplace Experience Measures with LGBT X Age Cohort Interaction Term (N = 37,219).

	Age Cohort Coefficient	LGBT Coefficient	Age Cohort * LGBT Coefficient	Age Cohort * LGBT *p*-value
*Treatment as Employee Models*				
Success fostered	0.014^†^	−0.155	0.021	0.672
Transparent evaluations	0.010	−0.052	−0.013	0.788
Adequate resources	0.006	−0.247^†^	0.042	0.413
Respected by supervisor	−0.004	−0.072	0.001	0.989
Diversity supported	0.001	−0.284	0.067	0.366
*Work Satisfaction Models*				
Personal satisfaction	0.014 **	−0.030	−0.009	0.705
Working conditions	0.026 ***	−0.133	0.026	0.574
Employee empowerment	0.039 ***	−0.116	0.004	0.978
Procedure satisfaction	0.030 ***	−0.149	0.006	0.915
Job satisfaction^^^	0.067 *	−0.376	−0.001	0.998

Note:

† *p* < 0.10,

* *p* < 0.05,

** *p* < 0.01,

*** *p* < 0.001 (two-tailed test).

^ indicates logit model; all other models are OLS regressions.

**Table 6.** OLS and Ordered Logistic Regression Models Predicting Workplace Experience Measures with LGBT Status, Run separately for Women and Men.

	MEN (N = 22,550)		WOMEN (N = 14,669)	
	LGBT coefficient	Std. Error	LGBT Coefficient	Std. Error
*Treatment as Employee Models*				
Success fostered	−0.119^†^	0.063	−0.099^†^	0.051
Transparent evaluations	−0.066	0.055	−0.112^†^	0.060
Adequate resources	−0.153 **	0.055	−0.177 **	0.069
Respected by supervisor	−0.061	0.044	−0.092	0.094
Diversity supported	−0.106^†^	0.053	−0.216^†^	0.127
*Work Satisfaction Models*				
Personal satisfaction	−0.049^†^	0.027	−0.045	0.073
Working conditions	−0.079	0.063	−0.087	0.112
Employee empowerment	−0.100 *	0.049	−0.136	0.086
Procedure satisfaction	−0.145 *	0.069	−0.130 **	0.046
Job satisfaction^^^	−0.456 *	0.196	−0.210	0.216

Note:

† *p* < 0.10,

* *p* < 0.05,

** *p* < 0.01,

*** *p* < 0.001 (two-tailed test).

^ indicates ordered logit model; all other models are OLS regressions.

## Footnotes

1 Along these lines, a recent Canadian study of pay gaps for gay men found that these gaps were smallest among public-sector workers and largest among private-sector workers [30].

2 We refer to these as “STEM-related agencies” rather than the more common “science agencies” because the former aligns more closely with the actual work done in these agencies (which includes mathematical, engineering, and technical work) and because “STEM” aligns with existing literature on inequality in technoscientific work.

3 The Energy Research and Development Agency was absorbed into the DOE upon its creation in 1977 [49].

4 For instance, in recent Pew polls, 76% of Republicans say that the government regulation of business does more harm than good (compared to 41% of Democrats) while 64% of Democrats (compared to only 28% of Republicans) believe that environmental protection should be strengthened [70].

5 Along similar lines, interviews with sexual minority graduate students in engineering suggested that they felt more comfortable being open about their sexual identity as teaching assistants than they were as undergraduate students [19].

6 FEVS was administered to a random sample of all permanent, non-seasonal employees of 37 large agencies and 45 independent agencies. The 2013 response rate was 48.2%, which is a typical response rate for workplace surveys [76].

7 The Stata “chained” command was used to produce the 20 imputed datasets. Seventeen percent of responses were missing on the diversity support measure and 16 percent were missing from the meritocratic organization measure. All other measures had less than 7 percent missing.

8 Because the sample of LGBT respondents at NSF is numerically small (N = 28), there may be LGBT*NSF effects that are too small to be picked up by the analysis. However, the p-values on the majority of the NSF*LGBT interaction terms are quite large, lending confidence that NSF does not, indeed, provide better workplace experiences than other agencies. The sample of LGBT respondents at NRC is similarly small. Several of the NRC*LGBT interaction terms approach full or marginal significance; with a larger sample, we may see an even more substantial NRC effect.

9 Older LGBT workers report marginally lower satisfaction with their pay than younger LGBT workers, possibly reflecting actual wage gaps for older LGBT employees that have accumulated over time.

10 The effects for employee satisfaction and job satisfaction are negative but do not reach statistical significance for women. However, the interaction term between LGBT *gender in supplemental analysis is nonsignificant, suggesting that LGBT-identifying women and men experience similar disadvantages on those measures.

11 Occupational segregation by gender likely plays a role in the results by gender as well. The sample of women includes STEM workers but also likely a larger proportion of non-STEM workers than among the sample of men. As such, these results likely underestimate biases that women working in STEM jobs in these agencies encounter.

12 We found that LGBT persons were equally as likely as their non-LGBT colleagues to have supervisory responsibilities in their agency.

## Appendix A

**Table A1.** Demographic Characteristics of STEM-Related Agencies (N = 37,219).

	Percent LGBT	Percent Women	Percent Racial/Ethnic Minorities	Mean, Tenure Measure	Mean, Age Cohort
NRC	2.8%	36.8%	30.5%	2.19	2.45
EPA	5.0%	54.7%	29.2%	2.47	2.47
DOE	2.7%	39.7%	22.8%	2.25	2.46
NSF	4.2%	64.5%	34.6%	2.34	2.58
NASA	2.2%	36.8%	24.3%	2.47	2.46
DOT	2.7%	32.0%	29.7%	2.29	2.56

**Table A2.** Unstandardized Coefficients (with Standard Errors) of LGBT X Agency Interaction Terms from OLS and Ordered Logistic Regression models Predicting Workplace Experience Measures (N = 37,219).

	EPA * LGBT Coef.	NRC * LGBT Coef.	NASA * LGBT Coef.	NSF * LGBT Coef.	DOT * LGBT Coef.	DOE * LGBT Coef.
	(*SE*, *p*-Value)	(*SE*, *p*-Value)	(*SE*, *p*-Value)	(*SE*, *p*-Value)	(*SE*, *p*-Value)	(*SE*, *p*-Value)
*Treatment as Employee Models*						
Success fostered	0.127 (0.089, *p* = 0.155)	0.143^†^ (0.084, *p* = 0.090)	0.095 (0.068, *p* = 0.164)	0.094 (0.145, *p* = 0.517)	−0.067 (0.092, *p* = 0.466)	−0.088 (0.081, *p* = 0.278)
Transparent evaluations	0.074 (0.083, *p* = 0.373)	0.178 * (0.089, *p* = 0.046)	0.048 (0.067, *p* = 471)	0.054 (0.127, *p* = 0.669)	−0.066 (0.089, *p* = 0.461)	−0.022 (0.075, *p* = 0.770)
Adequate resources	0.192 * (0.084, *p* = 0.023)	0.174^†^ (0.102, *p* = 0.090)	0.169 * (0.069, *p* = 0.014)	0.053 (0.154, *p* = 0.730)	−0.096 (0.091, *p* = 0.295)	−0.154 * (0.074, *p* = 0.038)
Respected by supervisor	0.059 (0.082, *p* = 0.475)	0.119 (0.078, *p* = 0.127)	0.026 (0.066, *p* = 0.695)	−0.033 (0.127, *p* = 0.795)	−0.065 (0.093, *p* = 0.483)	0.037 (0.075, *p* = 0.616)
Diversity supported	0.141 (0.101, *p* = 0.161)	0.255 ** (0.089, *p* = 0.004)	0.076 (0.095, *p* = 0.369)	0.108 (0.144, *p* = 0.453)	−0.128 (0.131, *p* = 0.330)	−0.036 (0.092, *p* = 0.698)
*Work Satisfaction Models*						
Personal satisfaction	0.084 (0.124, *p* = 0.497)	0.330 ** (0.119, *p* = 0.006)	0.023 (0.104, *p* = 0.821)	0.137 (0.181, *p* = 0.446)	−0.032 (0.148, *p* = 0.827)	−0.098 (0.109, *p* = 0.369)
Working conditions	0.152 * (0.068, *p* = 0.026)	0.115^†^ (0.066, *p* = 0.081)	0.009 (0.061, *p* = 0.883)	0.087 (0.099, *p* = 0.378)	−0.056 (0.093, *p* = 0.551)	−0.144 * (0.071, *p* = 0.041)
Employee empowerment	0.114 (0.088, *p* = 0.196)	0.143 (0.100, *p* = 0.153)	0.101 (0.070, *p* = 0.151)	0.292 * (0.131, *p* = 0.025)	−0.061 (0.093, *p* = 0.514)	−0.097 (0.081, *p* = 0.233)
Procedure satisfaction	0.174^†^ (0.092, *p* = 0.058)	0.170^†^ (0.093, *p* = 0.068)	0.076 (0.073, *p* = 0.302)	0.205 (0.128, *p* = 0.109)	−0.099 (0.103, *p* = 0.333)	−0.065 (0.081, *p* = 0.420)
Job satisfaction^^^	0.074 (0.106, *p* = 0.485)	0.080 (0.116, *p* = 0.492)	0.110 (0.084, *p* = 0.192)	0.093 (0.162, *p* = 0.564)	−0.047 (0.119, *p* = 0.695)	−0.065 (0.097, *p* = 0.502)

Note:

† *p* < 0.10,

* *p* < 0.05,

** *p* < 0.01,

*** *p* < 0.001 (two-tailed test).

^ indicates ordered logit model; all other models are OLS regressions.
